# Analysis of CT scan images for COVID-19 pneumonia based on a deep ensemble framework with DenseNet, Swin transformer, and RegNet

**DOI:** 10.3389/fmicb.2022.995323

**Published:** 2022-09-23

**Authors:** Lihong Peng, Chang Wang, Geng Tian, Guangyi Liu, Gan Li, Yuankang Lu, Jialiang Yang, Min Chen, Zejun Li

**Affiliations:** ^1^School of Computer Science, Hunan University of Technology, Zhuzhou, China; ^2^College of Life Sciences and Chemistry, Hunan University of Technology, Zhuzhou, China; ^3^Geneis (Beijing) Co., Ltd., Beijing, China; ^4^School of Computer Science, Hunan Institute of Technology, Hengyang, China

**Keywords:** COVID-19 pneumonia, CT scan image, deep ensemble, DenseNet, Swin transformer, RegNet

## Abstract

COVID-19 has caused enormous challenges to global economy and public health. The identification of patients with the COVID-19 infection by CT scan images helps prevent its pandemic. Manual screening COVID-19-related CT images spends a lot of time and resources. Artificial intelligence techniques including deep learning can effectively aid doctors and medical workers to screen the COVID-19 patients. In this study, we developed an ensemble deep learning framework, DeepDSR, by combining DenseNet, Swin transformer, and RegNet for COVID-19 image identification. First, we integrate three available COVID-19-related CT image datasets to one larger dataset. Second, we pretrain weights of DenseNet, Swin Transformer, and RegNet on the ImageNet dataset based on transformer learning. Third, we continue to train DenseNet, Swin Transformer, and RegNet on the integrated larger image dataset. Finally, the classification results are obtained by integrating results from the above three models and the soft voting approach. The proposed DeepDSR model is compared to three state-of-the-art deep learning models (EfficientNetV2, ResNet, and Vision transformer) and three individual models (DenseNet, Swin transformer, and RegNet) for binary classification and three-classification problems. The results show that DeepDSR computes the best precision of 0.9833, recall of 0.9895, accuracy of 0.9894, F1-score of 0.9864, AUC of 0.9991 and AUPR of 0.9986 under binary classification problem, and significantly outperforms other methods. Furthermore, DeepDSR obtains the best precision of 0.9740, recall of 0.9653, accuracy of 0.9737, and F1-score of 0.9695 under three-classification problem, further suggesting its powerful image identification ability. We anticipate that the proposed DeepDSR framework contributes to the diagnosis of COVID-19.

## Introduction

In December 2019, a novel acute atypical respiratory disease, COVID-19, has broken in Wuhan, China (Ksiazek et al., 2003; Zhou et al., 2020). COVID-19 was defined as a global pandemic by the World Health Organization on 3 November 2020. Till 26 June 2022, this disease has infected over 541 million individuals and caused over 6.3 million deaths (COVID Live—Coronavirus Statistics—Worldometer, 2022). COVID-19 has exacerbated human suffering, damaged the global economy, and seriously affected the health, environmental and social fields worldwide (Mofijur et al., 2021). It has still indirectly affected the global educational and religions level. Moreover, it has caused healthcare service resources to the brink in many countries and regions and will deeply affect medical research (Harper et al., 2020). Furthermore, middle-income countries especially low-income countries remain more vulnerable in preventing COVID-19 and need to face more serious challenges (Peters et al., 2020).

The COVID-19 pandemic has caused severe challenges to global public health (Wang et al., 2020; Sun et al., 2022a). The screening of massive samples each day overwhelms laboratories worldwide (Agaoglu et al., 2022). Detection of SARS-CoV-2 through RT-PCR from a nasopharyngeal swab sample is the most common avenue to diagnose COVID-19. However, RT-PCR does not demonstrate powerful sensitivity and specificity (Pu et al., 2022). Moreover, it need spend about 6 h for sampling and consecutive tests to distinguish false positives and false negatives (Lee et al., 2022). Multiple patients demonstrate clinical, laboratorial, and radiological features related to COVID-19, however, their RT-PCR test results are negative (Saad Menezes et al., 2022).

Many evidences have suggested that chest Computer Tomography (CT) is an accurate and efficient COVID-19 diagnosis avenue (Chung et al., 2020; Pan et al., 2020; Wang C C et al., 2021; Wang B et al., 2021). It has high sensitivity and low misdiagnosis rate, thus is an efficient complement to RT-PCR (Fields et al., 2021). Although it is vital to rapidly detect patients with the COVID-19 infection by CT images, expert thoracic radiologists are not likely to immediately diagnose positive cases at all times, which may not only cause treatment delay, but also urge further transmission of COVID-19 because the COVID-19 patients are not promptly isolated (Jin et al., 2020; Shorten et al., 2021; Afshar et al., 2022). In this situation, it is especially important to aid doctors and health care workers to distinguish COVID-19-related CT images from non-COVID-19-realted CT images using artificial intelligence techniques.

Many studies have suggested that artificial intelligence (AI) techniques including machine learning obtained enormous success in bioinformatics and medical image analysis (Chen et al., 2018a,b, 2019; Wang B et al., 2021; Wang C C et al., 2021; Zhang et al., 2021; Yang et al., 2022; Liu et al., 2022a). Over the last decade years, deep learning techniques have outperformed numerous state-of-the-art machine learning algorithms and demonstrated excellent learning ability in many fields including image recognition (Voulodimos et al., 2018; Wang B et al., 2021; Wang C C et al., 2021;Sun et al., 2022; Liu et al., 2022a,b).

Under the situation of no standardization, artificial intelligence technologies, especially deep learning, have been widely applied to data collection and performance evaluation for COVID-19 (Roberts et al., 2021). Abbas et al. (2021) proposed a novel convolutional neural network (CNN) model, DeTraC, to classify COVID-19-related chest X-ray images based on feature extraction, decomposition and class composition. Shalbaf and Vafaeezadeh (2021) designed a deep transfer learning-based ensemble model with different pre-trained CNN architectures to detect CT images for novel COVID-19 diagnosis. Zhang et al. (2020) developed a deep learning-based anomaly detection system to screen COVID-19 from chest x-ray images. Zhou et al. (2021) explored an ensemble deep learning framework to detect COVID-19 from CT images. Karbhari et al. (2021) introduced an auxiliary classifier generative adversarial network to generate synthetic chest X-ray images and further detect COVID-19 based on custom-made deep learning model. Chouat et al. (2022) exploited deep transfer learning algorithm to screen COVID-19 positive patients based on CT scan and chest X ray images. Fan et al. (2022) proposed a branch network model by combining CNN and transformer structure for the identification of COVID-19 using CT scan images. Ter-Sarkisov (2022) built a COVID-CT-Mask-Net model to diagnose COVID-19 through regional features from chest CT scan images. Chieregato et al. (2022) presented a deep learning-based COVID-19 prognostic hybrid model to support clinical decision making.

These models are mainly based on CNN and attention mechanism and effectively classify COVID-19-related images and non-COVID-19-related ones. However, they remain to improve the classification performance. In this study, we developed an ensemble deep learning framework (DeepDSR) by integrating three state-of-the-art neural networks including DenseNet, Swin transformer, and RegNet for the COVID-19 diagnosis.

## Materials and methods

### Materials

We use three available CT image datasets related to COVID-19 to investigate the performance of our proposed DeepDSR model. Dataset 1 can be downloaded from https://www.kaggle.com/datasets/plameneduardo/a-covid-multiclass-dataset-of-ct-scans. It contains publicly available 4,173 CT scan images from 210 different patients, out of which 2,168 images are from 80 patients infected by COVID-19 and confirmed by RT-PCR in hospitals from Sao Paulo, Brazil (Soares et al., 2020). Dataset 2 can be downloaded from https://www.kaggle.com/datasets/plameneduardo/sarscov2-ctscan-dataset. It contains 1,252 CT scan images from patients infected by COVID-19 and 1,230 CT scan images for patients non-infected by COVID-19 in hospitals from Sao Paulo, Brazil (Soares et al., 2020). Dataset 3 can be downloaded from https://github.com/UCSD-AI4H/COVID-CT. It contains 349 COVID-19 CT images from 216 patients and 463 non-COVID-19 CT images (Zhao et al., 2020).

**Figure 1:**
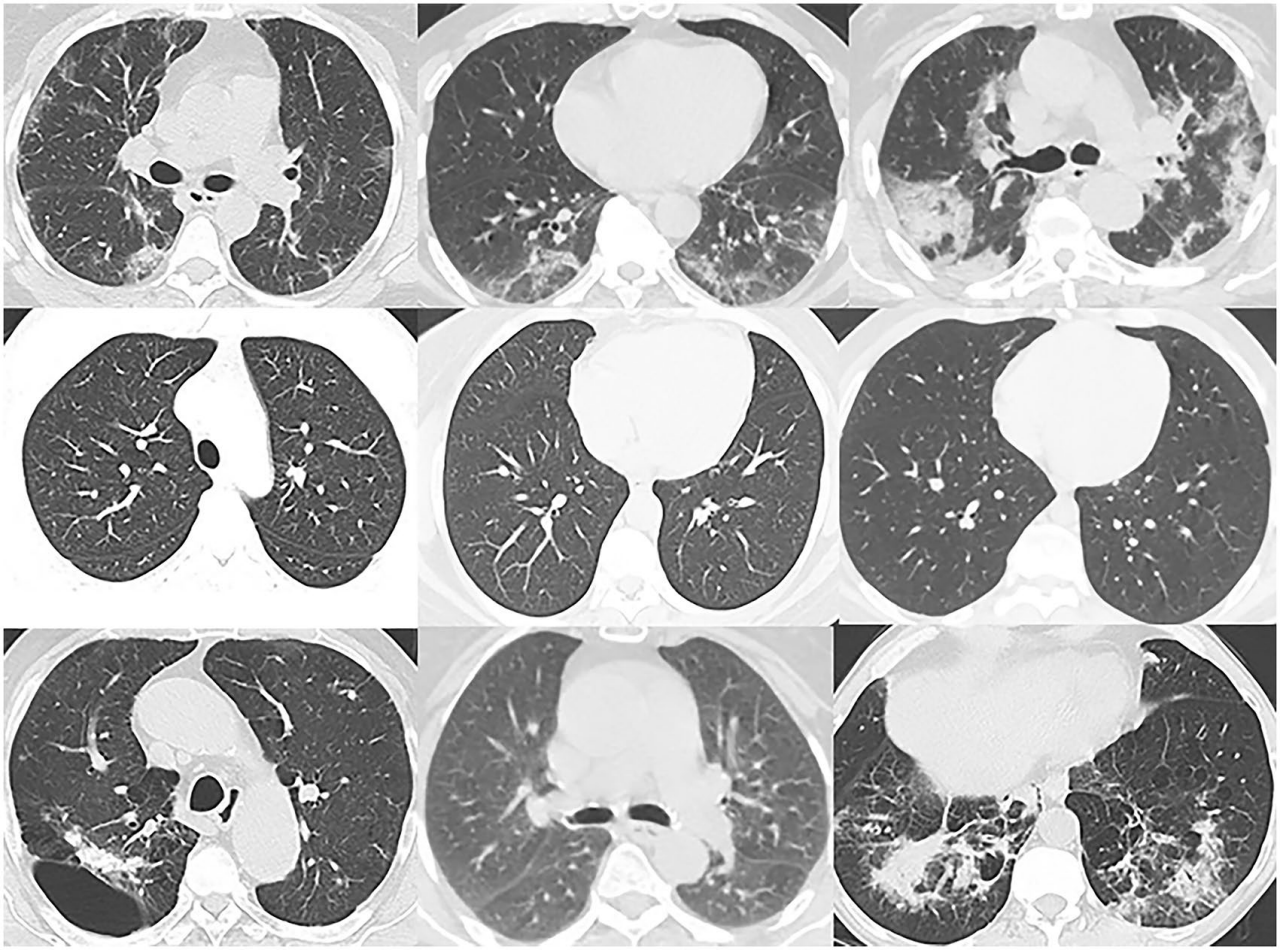
Image examples in dataset.

To boost the generalization ability of our proposed DeepDSR model, we integrate the above three datasets to one larger dataset. Consequently, DeepDSR can be used to effectively classify CT images in both individual datasets and other datasets. And we remove images with poor imaging and ones nonconforming to specifications. Finally, we obtain one dataset with 7,398 pulmonary CT images, which include 3,768 CT images from patients with the COVID-19 infections, 1,247 ones with other pneumonia infections, and 2,383 ones from normal lungs. We use 3,768 COVID-19-related images and 2,383 normal CT images to train the models for binary classification problems and use all 7,398 images for three classification problems. As shown in Figure 1, Lines 1–3 show pulmonary CT images from patients with COVID-19 infections, normal lungs, and patients with other pneumonia infections, respectively.

### The pipeline of DeepDSR

**Figure 2:**
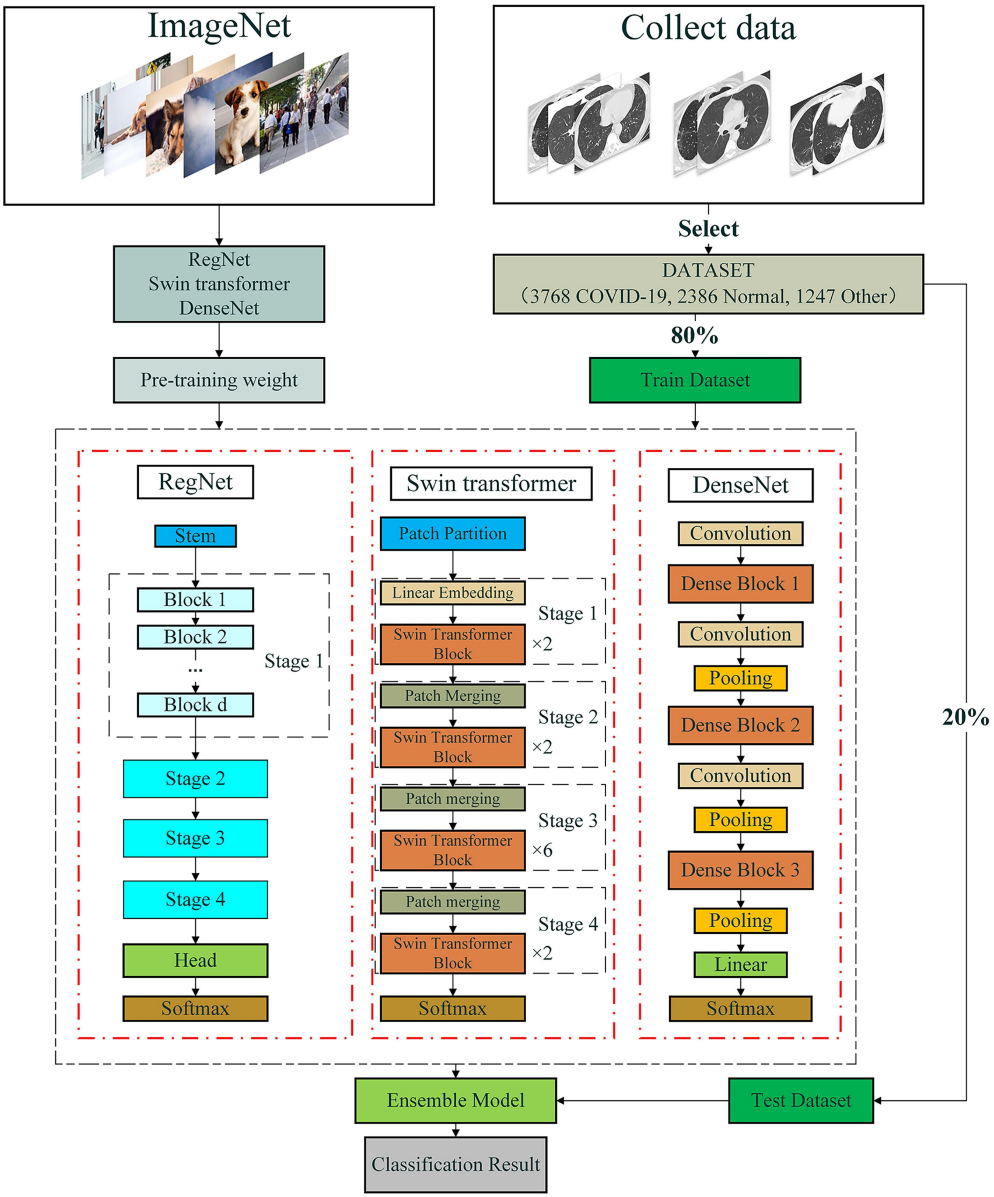
The pipeline for COVID-19-related CT image classification based on an ensemble of DenseNet, RegNet, and Swin transformer.

It is difficult to obtain the best prediction accuracy when only thousands of images are trained. Thus, we design an ensemble model to reduce the limitation of lack of data through transfer learning. The ensemble model integrates three state-of-the-art and different network architectures, that is, DenseNet, Swin transformer and RegNet. The pipeline is shown as Figure 2. As shown in Figure 2, first, we preprocess data by integrating three available COVID-19-related CT image datasets to one larger dataset. Second, we pretrain weights of DenseNet, Swin transformer, and RegNet on the ImageNet dataset based on transformer learning. Third, we continue to train DenseNet, Swin Transformer, and RegNet on the integrated larger dataset. Finally, the classification results are obtained by integrating results from the above three models and the soft voting approach.

### DenseNet

**Figure 3:**
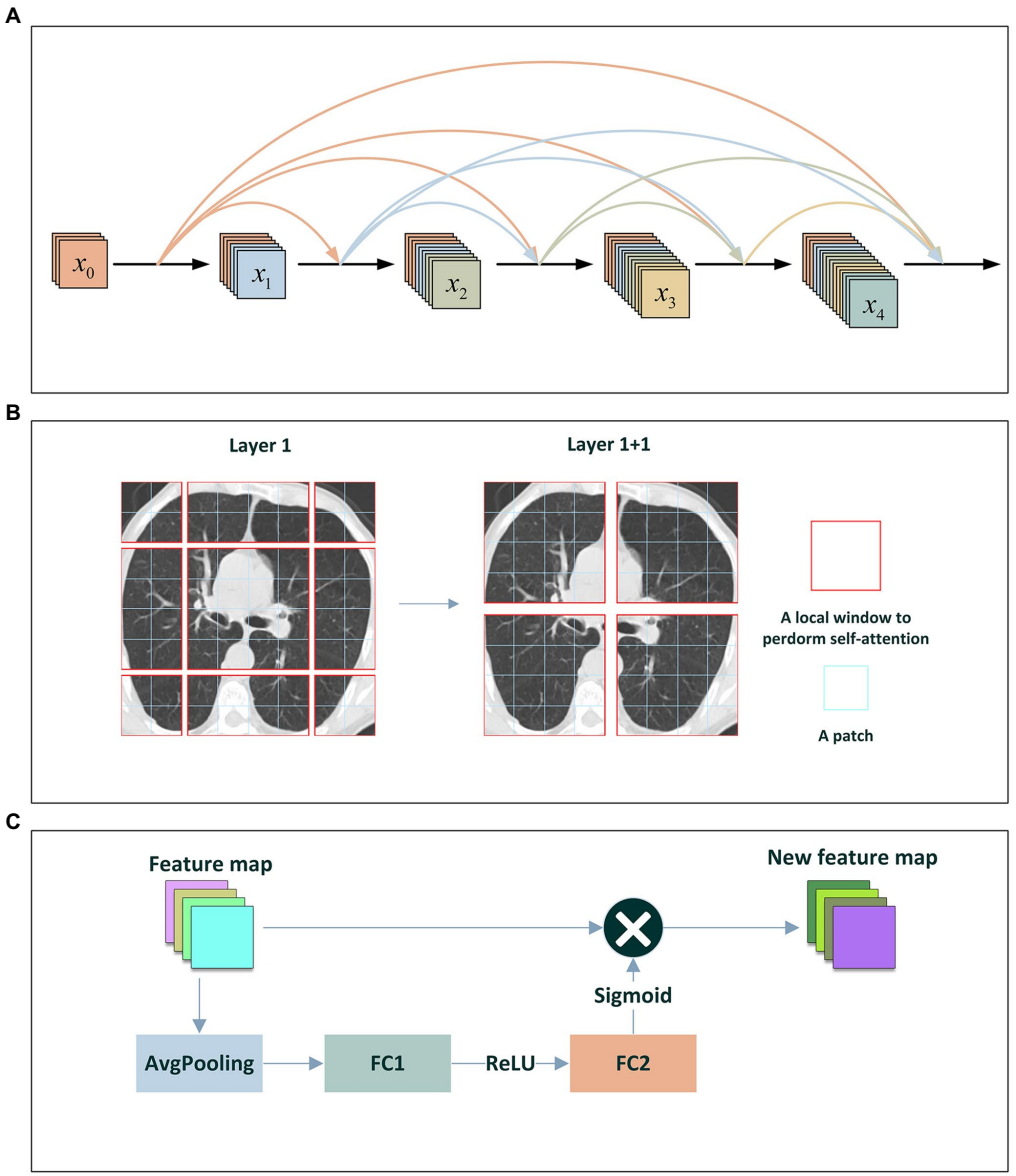
**(A)** The DenseNet Block; **(B)** Shifted-Window technique; **(C)** The Squeeze-and-Excitation network.

CNNs can implement accurate and efficient train when they contain shorter connections between layers close to the input and those close to the output. Traditional convolutional networks composed of L layers connect each layer to its subsequent layer. Inspired by the model proposed by Huang et al. (2017), we introduced a Dense convolutional Network, DenseNet, to classify COVID-19-related CT scan images. DenseNet implements connection between each layer in a feed-forward fashion to accurately and efficiently train the model. DenseNet with L layers has $\frac{1}{2}L\left(L+1\right)$ direct connections. At each layer, as shown in Figure 3A, the CT image feature maps from all previous layers are taken as its inputs and its outputs are taken as the inputs at next layer. For ResNet (Radosavovic et al., 2020), the original features and the new features are added by element by element to achieve the sample features. Differed from ResNet, DenseNet obtains shortcut through direct concatenation. DenseNet reduces the vanishing-gradient problem, boosts feature propagation, advances feature reuse while greatly decrease the number of parameters.

### Swin transformer

Transformer has difficulty in application from language to vision because of differences between the two areas. Thus, Liu et al. developed a hierarchical transformer to obtain data representation by shifted windows (Liu et al., 2021). For an image, first, transformer splits it into fixed-size patches. Second, the patches are linearly embedded and added position embeddings. Third, the embedded results are feed to a standard Transformer encoder. Finally, an extra learnable “classification token” is added to the sequence to classify images. Inspired by model proposed by Liu et al. (2021), we use the window-shift technique and design a Swin transformer to classify COVID-19-related CT scan images.

The window-shift technique and the sliding window approach are similar in modeling ability, but the former is beneficial for all-MLP architectures and has much lower latency than the latter. Swin transformer focuses on shifting window partition between consecutive self-attention layers. As shown in Figure 3B, the shifted windows connect with the windows in the previous layer, thus significantly enhancing the modeling ability. The window-shift technique limits self-attention computation to non-overlapping local windows as well as supports cross-window connection, thereby effectively improving the image classification ability of models. Furthermore, Swin transformer utilizes the window-shift technique and demonstrates the flexibility when modeling on COVID-19-related image identification as well as computational complexity linearly with image size.

### RegNet

Neural architecture search and RegNet are two representative neural network design paradigms. The two complementary design paradigms can improve the efficiency of search algorithms while develop better models. Neural architecture search mainly focuses on the search strategy to more efficiently find the best network instances in a fixed and manually designed search space. In contrast, RegNet (Radosavovic et al., 2020) more focuses on designing paradigms on novel design spaces.

RegNet is a novel neural network design paradigm. It used a residual network to simplify the deeper network training. It can boost the understanding of network design and further investigate design principles with strong generalize abilities across different settings. Instead of concentrating on individual network instance design, RegNet designs network design spaces that can parameterize network populations. The design process is similar to manually design network while advances the design space level. Consequently, we can obtain a low-dimensional design space composed of multiple simple and regular networks.

In this study, RegNet composes a stem with the stride of 2 and 32 3×3 convolution kernels, followed by a network body composed of a series of stages, and finally a head. In the network body, each stage operates at gradually reduced resolution. It consists of multiple identical blocks with the stride of 1 except that the first block uses stride-two convolution kernel. The head is composed of an average pooling layer and a fully connected layer. It is used to output n classes.

In addition, RegNet contains RegNetX and RegNetY composed of RegNetX and squeeze-and-excitation network. As shown in Figure 3C, the squeeze-and-excitation network generally composed of one global average pooling layer and two fully connection layers that separately use ReLU and sigmoid as activation functions.

### Ensemble

Although machine learning techniques achieve significant successes in knowledge discovery, they fail to obtain powerful performance because of imbalanced, high-dimensional and noisy features of data. Consequently, ensemble learning, which effectively integrates the prediction results from multiple individual classifiers, has been widely applied to image processing (Sagi and Rokach, 2018).

Ensemble learning methods first generate multiple weak predictive results using different machine learning models, and obtain better performance by ensemble of the results from each individual model with different voting strategies. It composes of five main types: bagging, AdaBoost, gradient boosting, random forest, and random sub-space (Dong et al., 2020). Bagging generates sample subsets based on the random sampling approach, and train basic learners in a parallel manner (Breiman, 1996). AdaBoost concentrates on improving classification ability of individual models *via* iteratively adjusting weights for all misclassified samples (Hastie et al., 2009). Gradient boosting achieves sample subsets based on the random sampling approach, and trains each classifier to alleviate the residuals caused by the previous model. Thus, gradient boosting better fits the real data (Friedman, 2002). Random forest takes decision trees as predictors and separately trains multiple models to reduce the overfitting problem (Breiman, 2001). Random subspace constructs a set of feature subspaces based on the random sampling approach, and trains learners on the feature subspace set. Finally, it obtains the final classification by combining the results from each individual classifier (Ho, 1995).

Ensemble learning utilizes different ensemble strategies to ensemble results from individual models. For regression estimation, it gains the final results *via* averaging all predictions. For classification, ensemble learning uses the voting method to achieve the final classification by combining each individual classifier. The absolute majority voting approach takes the same classification result as one from more than half of individual classifiers as the final result, and the relative majority voting approach takes the classification result where the number of individual predictors involved in a certain prediction is the largest as the final result. Therefore, we combine DenseNet, Swin transformer, and RegNet and develop an ensemble deep learning model, DeepDSR, to improve the COVID-19 classification performance of the model.

The classification scores from the three individual classifiers are integrated based on the soft voting approach. Given a query image, for a binary classification problem, suppose that its scores classified to COVID-19-related image by DenseNet, Swin transformer, and RegNet are $S_{1}^{\mathrm{covid}-19}$ , $S_{2}^{\mathrm{covid}-19}$ , and $S_{3}^{\mathrm{covid}-19}$ , respectively, its final score $S_{\mathrm{final}}^{\mathrm{covid}-19}$ classified to COVID-19-related sample can be represented by Eq. (1):


(1) $$S_{\mathrm{final}}^{\mathrm{covid}-19}=S_{1}^{\mathrm{covid}-19}+S_{2}^{\mathrm{covid}-19}+S_{3}^{\mathrm{covid}-19}$$

Similarly, its final score $S_{\mathrm{final}}^{non-\mathrm{covid}-19}$ classified to non-COVID-19-related image can be represented by Eq. (2):


(2) $$\begin{array}{l} S_{\mathrm{final}}^{non-\mathrm{covid}-19}=S_{1}^{non-\mathrm{covid}-19}+S_{2}^{non-\mathrm{covid}-19}\\ \;+S_{3}^{non-\mathrm{covid}-19}\; \end{array}$$

The image will be taken as COVID-19 related when $S_{\mathrm{final}}^{\mathrm{covid}-19}>S_{\mathrm{final}}^{non-\mathrm{covid}-19}$ , it will be taken as non-COVID-19 related, otherwise.

Furthermore, for a three-classification problem, suppose that its scores classified to COVID-19 related by DenseNet, Swin transformer, and RegNet are $S_{1}^{\mathrm{covid}-19}$ , $S_{2}^{\mathrm{covid}-19}$ , and $S_{3}^{\mathrm{covid}-19}$ , respectively, its final score $S_{\mathrm{final}}^{\mathrm{covid}-19}$ classified to positive sample can be computed by Eq. (3):


(3) $$S_{\mathrm{final}}^{\mathrm{covid}-19}=S_{1}^{\mathrm{covid}-19}+S_{2}^{\mathrm{covid}-19}+S_{3}^{\mathrm{pcovid}-19}$$

Similarly, its final score $S_{\mathrm{final}}^{\mathrm{other}}$ classified to other pneumonia can be computed by Eq. (4):


(4) $$S_{\mathrm{final}}^{\mathrm{other}}=S_{1}^{\mathrm{other}}+S_{2}^{\mathrm{other}}+S_{3}^{\mathrm{other}}$$

And its final score $S_{\mathrm{final}}^{\mathrm{normal}}$ from normal lung can be computed by Eq. (5):


(5) $$S_{\mathrm{final}}^{\mathrm{normal}}=S_{1}^{\mathrm{normal}}+S_{2}^{\mathrm{normal}}+S_{3}^{\mathrm{normal}}$$

Finally, the image will be taken as COVID-19 related when $S_{\mathrm{final}}^{\mathrm{covid}-19}$ is larger than $S_{\mathrm{final}}^{\mathrm{other}}$ and $S_{\mathrm{final}}^{\mathrm{normal}}$ ; it will be taken as other pneumonia when $S_{\mathrm{final}}^{\mathrm{other}}$ is larger than other two values; it is from normal lung otherwise.

### Transfer learning and pre-training

CNNs usually need to train a mass of parameters. However, it is almost impossible to learn such massive parameters only through a few training images (Zhuang et al., 2020; Zhu et al., 2021). In particular, transfer learning can utilize existing knowledge and transfer knowledge from source domains to the target domain and thus has been widely applied to solve problems in different while relevant fields (Pan and Yang, 2009; Weiss et al., 2016). It usually pretrains weights on a large-scale dataset using a standard neural architecture and then fine-tunes the weights on a target dataset. It has been successfully applied to medical image classification, for instance, cancer classification, pneumonia detection, and skin lesion identification (Chang et al., 2017; Deepak and Ameer, 2019; Khalifa et al., 2019; Chouhan et al., 2020).

Furthermore, existing lung CT scan images do not satisfy the need of a powerful image identification model because most of lung CT images are not publicly available. In addition, a image processed by random affine transformation, random crop or flip may not be a complete lung CT image because of the specificity of CT scanning techniques. The above two situations may easily produce the overfitting problem of image classification models in small datasets. Therefore, we want to pretrain the proposed DeepDSR model by transfer learning to advance the training speed, reduce overfitting, alleviate problems produced by insufficient data, and further improve the classification performance (Hijab et al., 2019; Cherti and Jitsev, 2021; Mustafa et al., 2021).

Finally, we developed an ensemble deep model (DeepDSR) to analyze COVID-19 CT images by combining DenseNet, Swin transformer, and RegNet. First, we integrate three COVID-19 image dataset to one larger dataset. Second, we pretrain weights of DenseNet, Swin Transformer, and RegNet on the ImageNet dataset. Third, we repeatedly select 80% of CT images from the integrated larger dataset as the training set and the remaining 20% as the test set. Fourth, the training set is used to train DenseNet, Swin transformer, and RegNet, respectively. The test set is used to test the performance of DenseNet, Swin transformer, and RegNet, respectively. Finally, the final classification results are obtained by integrating the results from the above three models.

## Results

### Experimental evaluation and parameter settings

To evaluate the performance of the proposed DeepDSR framework, we use six measurement metrics: precision, recall, accuracy, F1-score, AUC and AUPR. Suppose that True Positive (TP), True Negative (TN), False Negative (FN), and False Negative (FN) are defined as Table 1. We can compute precision, recall, accuracy, F1-score, True Positive Rate (TPR), and False Positive Rate (FGR) as follows:


(6) $$\mathrm{Precision}=\frac{TP}{TP+FP}$$

(7) $$\mathrm{Recall}=\frac{TP}{TP+FN}$$

(8) $$\mathrm{Accuracy}=\frac{TP+TN}{TP+TN+FP+FN}$$

(9) $$F_{1}-\mathrm{score}=\frac{2\cdot \mathrm{Precision}\cdot \mathrm{Recall}}{\mathrm{Precision}+\mathrm{Recall}}$$

(10) $$TPR=\frac{TP}{TP+FN}$$

(11) $$FPR=\frac{FP}{TN+FP}$$

**Table 1:** The confusion matrix.

		True results	
		Positive	Negative
Predicted results	Positive	TP	FP
	Negative	FN	TN

And AUC is the area under the TPR-FPR curve, and AUPR is the area under the precision-recall curve. For each sample (image), its classification scores from three individual networks (DenseNet, Swin transformer, and RegNet) are computed by the softmax layer, respectively. Its final classification probability is obtained by averaging the scores from the three single models. AUC and AUPR can be computed based on the obtained final classification probability.

Moreover, the six metrics are not equally important to COVID-19 CT image classification. The results caused by false negatives are more severe than ones caused by false positives for medical image classification. Therefore, recall and AUPR are more important compared to the other four evaluation metrics.

The experiments are performed for 100 epochs to obtain the optimal parameter settings. In addition, DenseNet and RegNet use stochastic gradient descent algorithm and Swim transformer uses AdamW as optimizer to update parameters. The detailed parameters are set in Table 2. In Table 2 and the following Tables 2–5, the bold font in each column represents the best performance computed by corresponding method.

**Table 2:** Parameter settings.

Model	Parameter setting
Swin transformer	epochs = 100, batch_size = 8, lr = 0.0001
RegNet	epochs = 100, batch_size = 16, lr = 0.001, lrf = 0.01
DenseNet	epochs = 100, batch_size = 16, lr = 0.001, lrf = 0.01

**Table 3:** The performance comparison of DeepDSR and other models for COVID-19 image binary classification.

	Precision	Recall	Accuracy	F1-score	AUC	AUPR
EfficientNetV2	0.5077	0.9015	0.6231	0.6495	0.7800	0.6649
ResNet	0.9786	0.9602	0.9764	0.9693	0.9960	0.9943
Vision transformer	0.9811	0.9769	0.9838	0.9790	0.9982	0.9975
DeepDSR	**0.9833**	**0.9895**	**0.9894**	**0.9864**	**0.9991**	**0.9986**

The bold fonts represent the best performance in each column.

**Table 4:** The performance comparison of DeepDSR and three individual models for binary classification problem.

	Precision	Recall	Accuracy	F1-score	AUC	AUPR
Swin transformer	0.9619	0.9539	0.9675	0.9579	0.9943	0.9924
RegNet	0.9571	0.9832	0.9764	0.9700	0.9963	0.9949
DenseNet	0.9770	0.9790	0.9829	0.9780	0.9981	0.9973
DeepDSR	**0.9833**	**0.9895**	**0.9894**	**0.9864**	**0.9991**	**0.9986**

The bold fonts represent the best performance in each column.

**Table 5:** Statistical analyses of four models on 1,231 images.

	DenseNet	Swin transformer	RegNet	DeepDSR
TN	743	736	733	**746**
FN	10	22	8	**5**
FP	11	18	21	**8**
TP	467	455	469	**472**

The bold fonts represent the best performance in each column.

### The performance comparison of DeepDSR with other three models for COVID-19 image binary classification

We compare the proposed DeepDSR method to three state-of-the-art deep learning models (efficientNetV2, ResNet, and Vision transformer) when classifying CT scan images to two classes: COVID-19 related or non-COVID-19 related. EfficientNetV2 (Tan and Le, 2021) aims to solve the problem of slow training when the size of the training image is large in efficientNetV1. Moreover, it replaced some MBConv structures in shallow layers with Fused-MBConv structures and found the optimal combination through neural architecture search technology to improve the network training speed. Finally, efficientNetV2 used a non-uniform scaling strategy to scale the model and thus make the model more reasonable.

ResNet (He et al., 2016) aims to solve the vanishing gradient and network degradation problems in traditional neural networks. ResNet solved the vanishing gradient problem through data preprocessing and batch normalization layer, and reduced the network degradation problem through a residual structure. ResNet used a connection model of shortcut to add interlayers in the feature matrix and thus greatly improve the depth of the network.

Transformer (Vaswani et al., 2017) has been broadly used in the natural language processing field. Attention mechanism has been widely used in the computer vision field. Inspired by the transformer mechanism, Vaswani et al. divided each image into patches, and took the linear embedded sequence of these image blocks as the input of the transformer. The processing method of image patches is the same as marks in NLP applications. Vision transformer (Dosovitskiy et al., 2020) achieved excellent results when both pretraining on a sufficient scale dataset and migrating to tasks with fewer data points.

**Figure 4:**
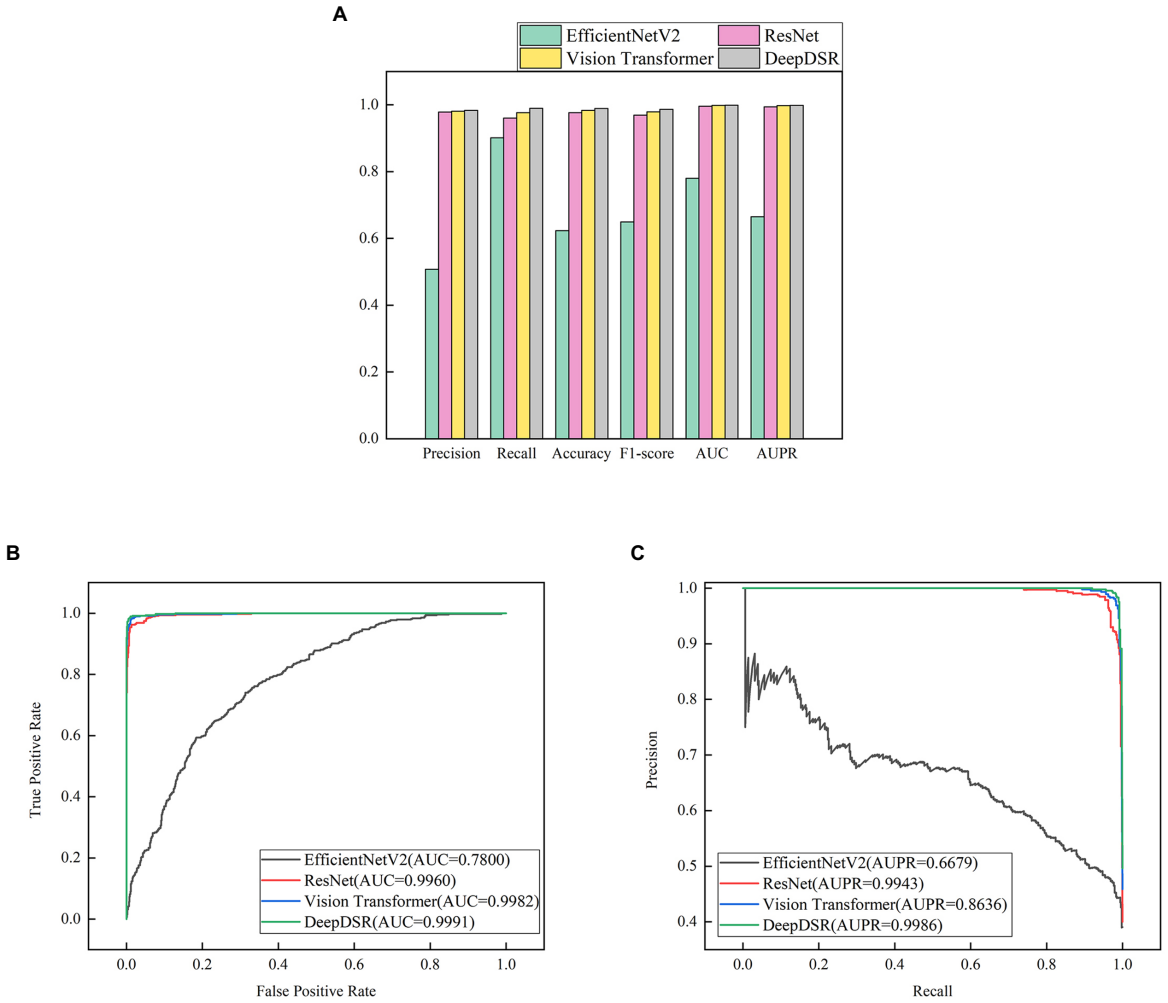
**(A)** The performance comparison of DeepDSR and other models for COVID-19 image binary classification. **(B,C)** The AUC and AUPR values of DeepDSR and other models for COVID-19 image binary classification.

We first selected 80% images as training set and 20% as test set from the integrated COVID-19-related CT scan images. We then train DeepDSR, efficientNetV2 (Tan and Le, 2021), ResNet (He et al., 2016), and Vision transformer (Dosovitskiy et al., 2020) for 100 epochs, respectively. The results are shown in Table 3 and Figure 4A. We can find that DeepDSR significantly outperforms efficientNetV2 in terms of precision, recall, accuracy, F1-score, AUC and AUPR. For examples, DeepDSR outperforms 21.93% and 33.42% compared to efficientNetV2 based on AUC and AUPR, respectively. DeepDSR also performs better than ResNet and Vision transformer although the improvement is slight. Figures 4B,C illustrate the AUC and AUPR values of DeepDSR and other models when classifying COVID-19-related CT images to two classes. The above results show that DeepDSR can efficiently identify CT scan images for patients infected by COVID-19.

### The performance comparison of DeepDSR and three individual models for COVID-19 image binary classification

**Figure 5:**
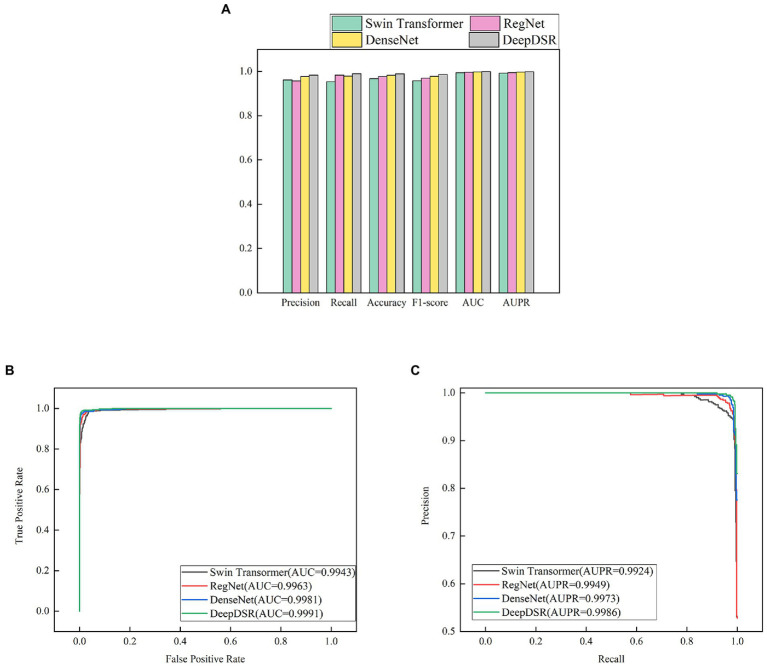
**(A)** The performance comparison of DeepDSR and three individual models for COVID-19 binary classification problem; **(B,C)** The AUC and AUPR values of DeepDSR and three individual models for COVID-19 binary classification problem.

To investigate the image classification performance of the proposed DeepDSR model with DenseNet, Swin transformer, and RegNet, we conduct experiment for 100 epochs. At each epoch, we select 80% samples to train DeepDSR, DenseNet, Swin transformer, and RegNet and the remaining 20% to test their performance. Table 4 and Figure 5A demonstrate the prediction results of the above four models. The results show that the proposed ensemble model, DeepDSR, outperforms other three individual models in terms of precision, recall, accuracy, F1-score, AUC, and AUPR. Figures 5B,C illustrate the AUC and AUPR values obtained from the above four models. We find that DeepDSR, ensemble of DenseNet, Swin transformer, and RegNet, can more effectively classify CT images to two classes: COVID-19-related or not.

### Statics of true positives/negatives and false positives/negatives

**Figure 6:**
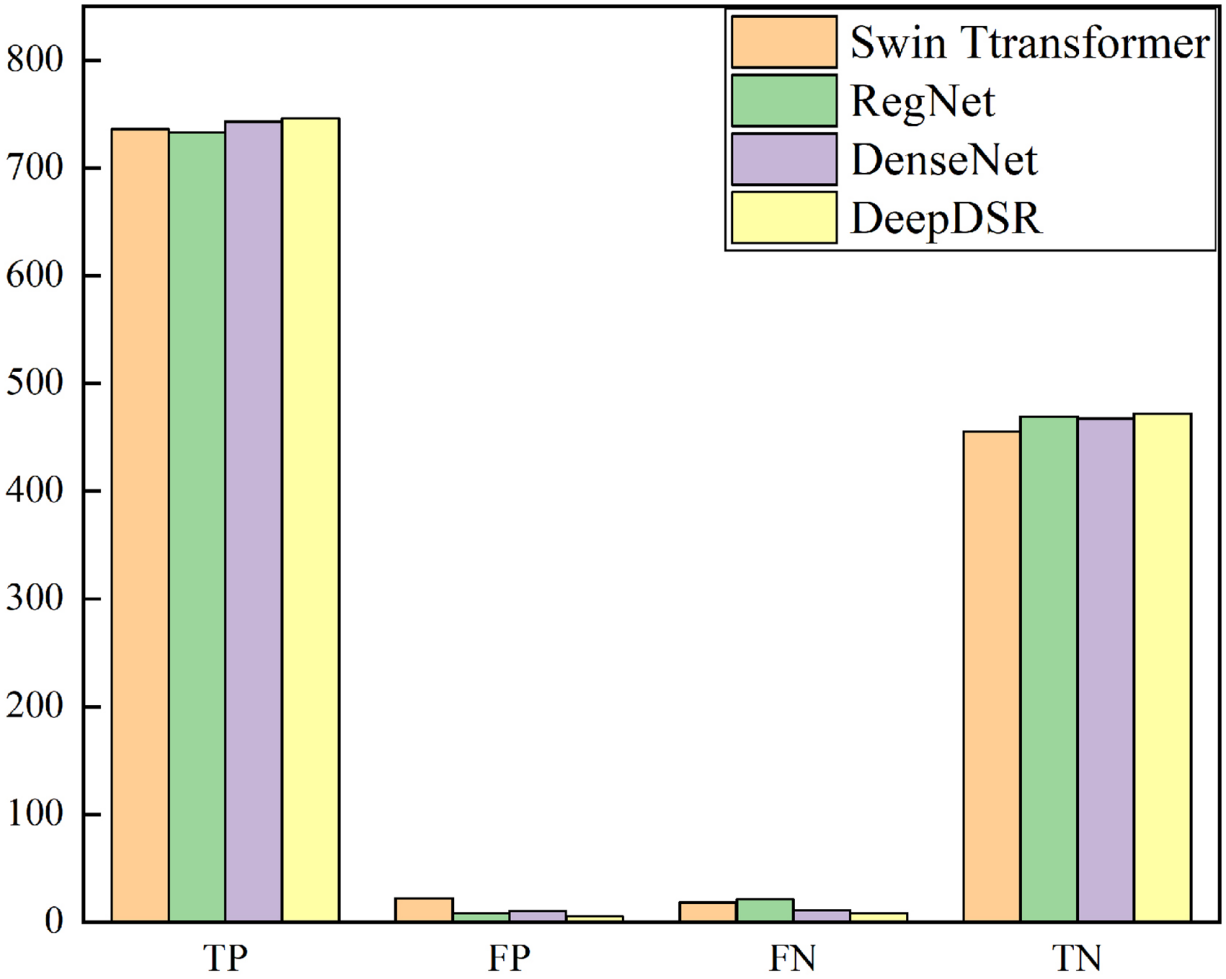
Statistical analysis of four methods on 1,231 images.

We investigate the classification results on 1,231 COVID-19-related CT images from the test set to more intuitively illustrate the affect of DeepDSR on CT image identification performance. Table 5 and Figure 6 give the number of true positives (TP), false positives (FP), false negatives (FN), and true negatives (TN) computed by DeepDSR, DenseNet, Swin transformer, and RegNet, respectively.

The results show that DeepDSR, DenseNet, Swin transformer, and RegNet misclassify a few samples. DeepDSR computes the most TPs and TNs while the least FPs and FNs. Furthermore, efficientNetV2, ResNet, and Vision transformer compute much more FPs and FNs compared with DeepDSR, demonstrating higher error rates. Moreover, DeepDSR, ensemble of DenseNet, Swin transformer, and RegNet, outperforms all other three individual models. Thus, the neural network, combining the predictions obtained from all the base models, can significantly improve the CT image classification performance of models. In addition, the stacking ensemble consisting of all three base models outperforms all other combinations. DeepDSR is tuned to utilize those predictions that help improve the classification performance and ignore the wrong predictions made by the base models.

### The affect of transfer learning on the performance

**Figure 7:**
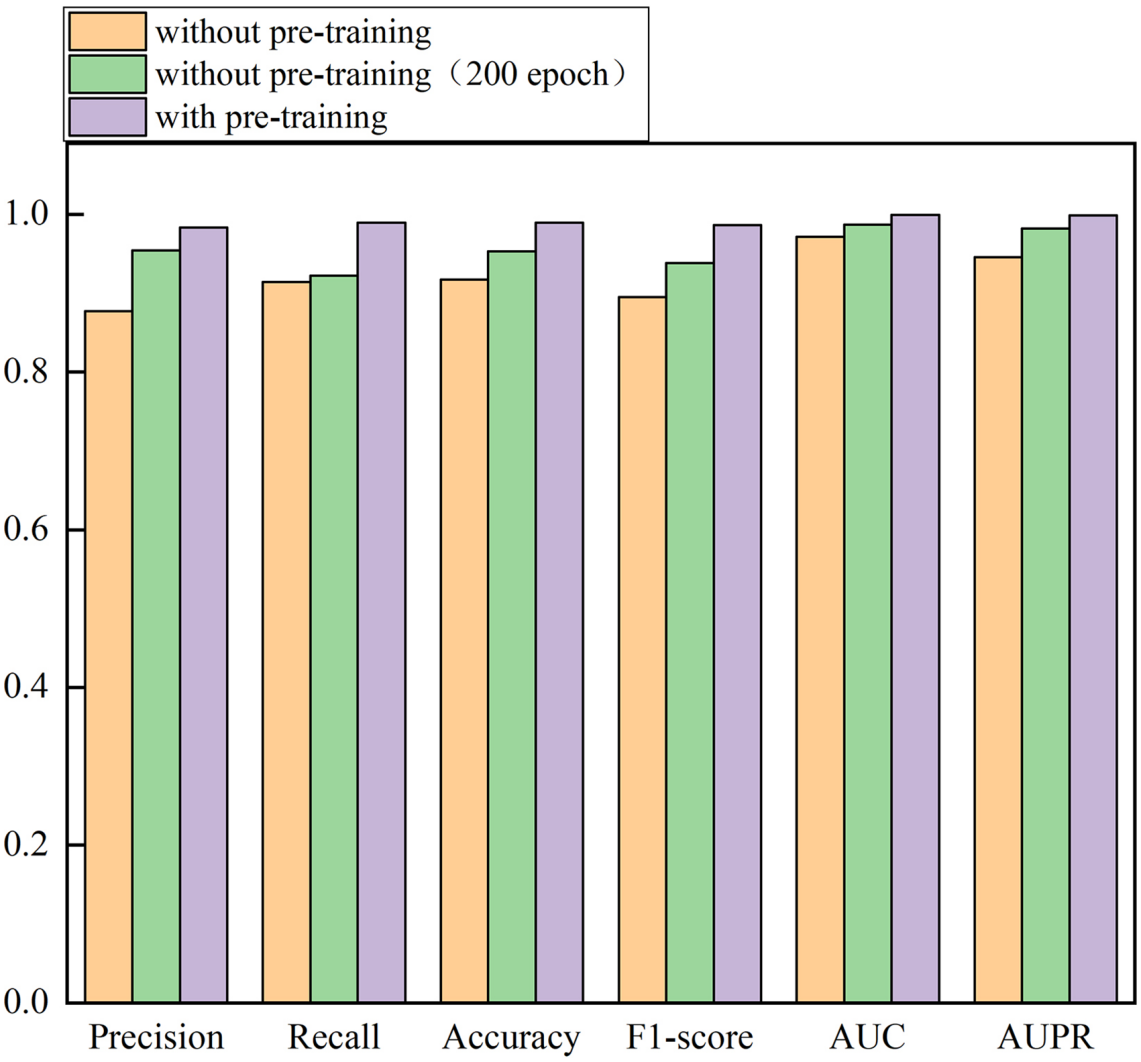
The affect of transfer learning on the performance.

In the above sections, we pretrain the weights of DenseNet, Swin transformer, and RegNet on the ImageNet dataset and continue to train the three models on the integrated larger dataset for 100 epochs. We set up a group of control experiments without pretraining (100 epochs and 200 epochs) to validate the importance of pretraining weights of the models for 100 epochs. The results are shown in Table 6 and Figure 7.

**Table 6:** The affect of transfer learning on the performance.

	Precision	Recall	Accuracy	F1-score	AUC	AUPR
With pre-train	**0.9833**	**0.9895**	**0.9894**	**0.9864**	**0.9991**	**0.9986**
Without pre-train	0.8773	0.914	0.9171	0.8953	0.9716	0.9455
Without pre-train (200 epoch)	0.9544	0.9224	0.9529	0.9382	0.9866	0.9821

The bold fonts represent the best performance in each column.

From Table 6 and Figure 7, we can observe that the performance of network architecture with the pretrained weights is much better than that of the network without pretraining weights for 100 epochs and 200 epochs. For example, under 100 epochs, the pretrained network computes accuracy of 0.9894, AUC of 0.9991, and AUPR of 0.9986, outperforming 7.88%, 2.83%, and 5.61% than the network without pretraining, respectively. In addition, we also investigate the performance of DeepDSR with pretraining for 100 epochs and ones without pretraining for 200 epochs. The results show that the pretrained network significantly outperforms the network without pretraining even for 200 epochs. Accuracy, AUC, and AUPR computed by the pretrained network are better 3.83%, 1.27%, and 1.68% than ones without pretraining for 200 epochs, respectively. The results demonstrate that pretraining based on transfer learning can reduce the training time while improve the classification performance. Finally, when adding epochs on the pretrained network, however, the performance improvement is not obvious. On the contrary, it even produces drifts and thus causes poorer performance.

### Performance comparison for three-classification problem

**Figure 8:**
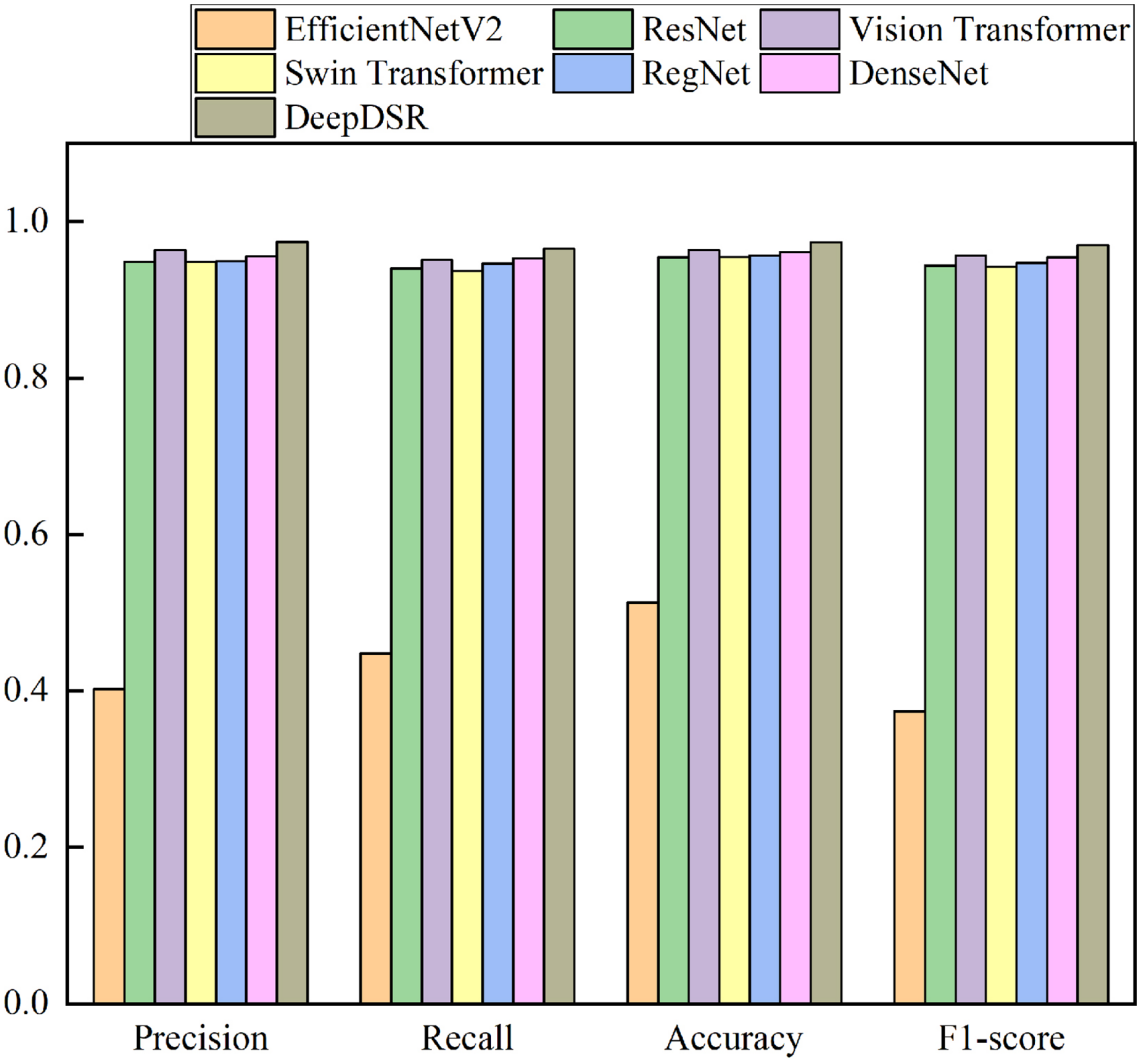
The performance of DeepDSR and other models for three-classification problem.

Finally, we classify CT scan images to three classes to further evaluate the robustness and credibility of DeepDSR. We use 7,398 lung CT scan images, which contain 3,768 lung CT scan images from patients infected by COVID-19, 2,383 ones from normal lung, and 1,247 ones from patients infected by other pneumonia. And 80% images are selected the training set and the remaining images are the test set. We repeatedly conduct the three-classification experiments on the obtained 7,398 images for 100 epochs. Table 7 and Figure 8 give precision, recall, accuracy, and F1-socre of DeepDSR, other three comparative methods, and three individual models.

**Table 7:** The performance of DeepDSR and other models for three-classification problem.

	Precision	Recall	Accuracy	F1-score
EfficientNet V2	0.4023	0.4479	0.5132	0.3736
ResNet	0.9487	0.9397	0.9541	0.9439
Vision transformer	0.7112	0.6264	0.7373	0.6301
Swin transformer	0.9488	0.9371	0.9548	0.9424
RegNet	0.9492	0.9463	0.9568	0.9476
DenseNet	0.9552	0.953	0.9608	0.9541
DeepDSR	**0.974**	**0.9653**	**0.9737**	**0.9695**

The bold fonts represent the best performance in each column.

The results from Table 7 and Figure 8 show that the proposed DeepDSR framework significantly outperforms efficientNet-V2 and Vision transformer in terms of precision, recall, accuracy, and F1-score. DeepDSR is also better than ResNet and three individual models based on the above measurement metrics. For example, DeepDSR computes the best precision of 0.9740, recall of 0.9653, accuracy of 0.9737, and F1-score of 0.9695, outperforming 1.93%, 1.27%, 1.31%, and 1.59 compared the second-best methods (DenseNet, DenseNet, RegNet, and DenseNet), respectively. The results demonstrate that DeepDSR has better generalization ability and can thus be applied to classify COVID-19-related CT scan images.

## Conclusion

COVID-19 detection through CT scan images has the characteristics of high sensitivity, low misdiagnosis rate, and high commercial practicability. Therefore, it has been a research hotspot to detect COVID-19 through CT scan images based on deep learning. In this study, we developed a deep ensemble model, DeepDSR to identify CT scan images for patients infected by COVID-19. DeepDSR combined three different state-of-the-art network architectures, DenseNet, Swin transformer, and RegNet. It obtained the best performance compared to three classical deep learning models (efficientNetV2, ResNet, and Vision transformer) as well as three individual models when classifying CT images to two classes (COVID-19-related or non-COVID-19-related) or three classes (COVID-19-related, normal pneumonia, and healthy lung).

EfficientNetV2, ResNet, and Vision transformer are three state-of-the-art deep learning models with different network architectures. The proposed DeepDSR model computed the best measurement values compared with the three network architectures, demonstrating its optimal image classification ability. Moreover, DeepDSR aggregated three individual deep models, DenseNet, Swin transformer, and RegNet. Lower correlations between the three individual models more obviously reduced the variance of DeepDSR. In addition, DeepDSR also reduced its variance due to the ensemble nature. Therefore, DeepDSR, ensemble of different single models, significantly outperforms the three individual models, thereby suggesting its powerful performance.

Our proposed DeepDSR has three advantages: first, three COVID-19-related CT image datasets were fused to boost the generalization ability of DeepDSR. Moreover, multiple methods including batch normalization were adopted to prevent overfitting. Finally, DeepDSR, ensemble of DenseNet, Swin transformer, and RegNet, can more accurately classify CT images and thus improve the classification performance. However, the training of DeepDSR was more complex than single model, it also spend more time to train and test the model, and more parameters need to be adjusted, thereby requiring more computing resources. In the future, we will design more robust ensemble deep learning models to accurately classify images for query diseases including COVID-19 and cancer. In particular, we will further consider deep heterogeneous ensemble framework to accurately identify images for related diseases by ensemble of deep learning model and supervised learning model.

## Data availability statement

The original contributions presented in the study are included in the article/supplementary material. Source code is freely downloadable at: https://github.com/plhhnu/DeepDSR/. Datasets 1-3 can be downloaded from the following three links: https://www.kaggle.com/datasets/plameneduardo/a-covid-multiclass-dataset-of-ct-scans; https://www.kaggle.com/datasets/plameneduardo/sarscov2-ctscan-dataset; https://github.com/UCSD-AI4H/COVID-CT.

## Author contributions

LP, CW, MC, and ZL: conceptualization. LP, CW, and ZL: methodology. CW and MC: software. LP, CW, GT, GLiu: validation. LP, MC, and ZL: investigation. CW, GLi, and YL: data curation. LP and CW: writing—original draft preparation. LP, GT, and JY: writing—review and editing. LP: supervision. LP, CW, and MC: project administration. LP, and MC: funding acquisition. All authors contributed to the article and approved the submitted version.

## Funding

ZL was supported by National Natural Science Foundation of China under grant no. 62172158. LP was supported by the National Natural Science Foundation of China under grant no. 61803151. GLiu and YL were supported by the Innovation and Entrepreneurship Training Program for College Students of Hunan Province under grant no. S202111535031 and the Innovation and Entrepreneurship Training Program for College Students of Hunan University of Technology under grant no. 20408610119.

## Conflict of interest

GT and JY were employed by Geneis (Beijing) Co. Ltd.

The remaining authors declare that the research was conducted in the absence of any commercial or financial relationships that could be constructed as a potential conflict of interest.

## Publisher’s note

All claims expressed in this article are solely those of the authors and do not necessarily represent those of their affiliated organizations, or those of the publisher, the editors and the reviewers. Any product that may be evaluated in this article, or claim that may be made by its manufacturer, is not guaranteed or endorsed by the publisher.

## References

[ref1] Abbas A. Abdelsamea M. M. Gaber M. M. (2021). Classification of COVID-19 in chest X-ray images using DeTraC deep convolutional neural network. Appl. Intell. 51, 854–864. doi: 10.1007/s10489-020-01829-7, PMID: 34764548 PMC7474514

[ref2] Afshar P. Rafiee M. J. Naderkhani F. Heidarian S. Enshaei N. Oikonomou A.. (2022). Human-level COVID-19 diagnosis from low-dose CT scans using a two-stage time-distributed capsule network. Sci. Rep. 12, 1–11. doi: 10.1038/s41598-022-08796-8 35318368 PMC8940967

[ref3] Agaoglu N. B. Yildiz J. Dogan O. A. Kose B. Alkurt G. Demirkol Y. K.. (2022). COVID-19 PCR test performance on samples stored at ambient temperature. J. Virol. Methods 301:114404. doi: 10.1016/j.jviromet.2021.114404, PMID: 34921841 PMC8673954

[ref4] Breiman L. (1996). Bagging predictors. Mach. Learn. 24, 123–140. doi: 10.1007/BF00058655

[ref5] Breiman L. (2001). Random forests. Mach. Learn. 45, 5–32. doi: 10.1023/A:1010933404324

[ref6] Chang J. Yu J. Han T. Chang H. J. Park E. (2017). A method for classifying medical images using transfer learning: A pilot study on histopathology of breast cancer. In *2017 IEEE 19th international conference on e-health networking, applications and services (Healthcom)* (pp. 1–4). IEEE.

[ref7] Chen X. Xie D. Wang L. Zhao Q. You Z. H. Liu H. (2018b). BNPMDA: bipartite network projection for MiRNA–disease association prediction[J]. Bioinformatics 34, 3178–3186. doi: 10.1093/bioinformatics/bty333, PMID: 29701758

[ref8] Chen X. Yin J. Qu J. Huang L. (2018a). MDHGI: matrix decomposition and heterogeneous graph inference for miRNA-disease association prediction[J]. PLoS Comput. Biol. 14:e1006418. doi: 10.1371/journal.pcbi.1006418, PMID: 30142158 PMC6126877

[ref9] Chen X. Zhu C. C. Yin J. (2019). Ensemble of decision tree reveals potential miRNA-disease associations[J]. PLoS Comput. Biol. 15:e1007209. doi: 10.1371/journal.pcbi.1007209, PMID: 31329575 PMC6675125

[ref10] Cherti M. Jitsev J. (2021). Effect of Pre-Training Scale on Intra-and Inter-Domain Full and Few-Shot Transfer Learning for Natural and Medical X-Ray Chest Images. arXiv [Preprint] arXiv:2106.00116.

[ref11] Chieregato M. Frangiamore F. Morassi M. Baresi C. Nici S. Bassetti C.. (2022). A hybrid machine learning/deep learning COVID-19 severity predictive model from CT images and clinical data. Sci. Rep. 12, 1–15. doi: 10.1038/s41598-022-07890-1 35288579 PMC8919158

[ref12] Chouat I. Echtioui A. Khemakhem R. Zouch W. Ghorbel M. Hamida A. B. (2022). COVID-19 detection in CT and CXR images using deep learning models. Biogerontology 23, 65–84. doi: 10.1007/s10522-021-09946-7, PMID: 35064446 PMC8782709

[ref13] Chouhan V. Singh S. K. Khamparia A. Gupta D. Tiwari P. Moreira C.. (2020). A novel transfer learning based approach for pneumonia detection in chest X-ray images. Appl. Sci. 10:559. doi: 10.3390/app10020559

[ref14] Chung M. Bernheim A. Mei X. Zhang N. Huang M. Zeng X.. (2020). CT imaging features of 2019 novel coronavirus (2019-nCoV). Radiology 295, 202–207. doi: 10.1148/radiol.2020200230, PMID: 32017661 PMC7194022

[ref15] Deepak S. Ameer P. M. (2019). Brain tumor classification using deep CNN features via transfer learning. Comput. Biol. Med. 111:103345. doi: 10.1016/j.compbiomed.2019.103345, PMID: 31279167

[ref16] Dong X. Yu Z. Cao W. Shi Y. Ma Q. (2020). A survey on ensemble learning. Front. Comp. Sci. 14, 241–258. doi: 10.1007/s11704-019-8208-z

[ref17] Dosovitskiy A. Beyer L. Kolesnikov A. Weissenborn D. Zhai X. Unterthiner T.. (2020). An image is worth 16x16 words: transformers for image recognition at scale. arXiv [Preprint] arXiv:2010.11929.

[ref18] Fan X. Feng X. Dong Y. Hou H. (2022). COVID-19 CT Image Recognition Algorithm based on Transformer and CNN. Displays 72:102150. doi: 10.1016/j.displa.2022.102150 35095128 PMC8785369

[ref19] Fields B. K. Demirjian N. L. Dadgar H. Gholamrezanezhad A. (2021). Imaging of COVID-19: CT, MRI, and PET. Semin. Nucl. Med. 51, 312–320. doi: 10.1053/j.semnuclmed.2020.11.003 33288215 PMC7703471

[ref20] Friedman J. H. (2002). Stochastic gradient boosting. Comput. Stat. Data Analy. 38, 367–378. doi: 10.1016/S0167-9473(01)00065-2

[ref21] Harper L. Kalfa N. Beckers G. M. A. Kaefer M. Nieuwhof-Leppink A. J. Fossum M.. (2020). The impact of COVID-19 on research[J]. J. Pediatr. Urol. 16, 715–716. doi: 10.1016/j.jpurol.2020.07.002, PMID: 32713792 PMC7343645

[ref22] Hastie T. Rosset S. Zhu J. Zou H. (2009). Multi-class adaboost. Statistics and its. Interface 2, 349–360. doi: 10.4310/SII.2009.v2.n3.a8

[ref23] He K. Zhang X. Ren S. Sun J. (2016). Deep residual learning for image recognition. In *Proceedings of the IEEE conference on computer vision and pattern recognition* (pp. 770–778).

[ref24] Hijab A. Rushdi M. A. Gomaa M. M. Eldeib A. (2019). Breast cancer Classification in Ultrasound Images using Transfer Learning. In *2019 Fifth international conference on advances in biomedical engineering (ICABME)* (pp. 1–4). IEEE.

[ref25] Ho T. K. (1995). Random Decision Forests. In *Proceedings of 3rd international conference on document analysis and recognition* (*Vol. 1*, pp. 278–282). IEEE.

[ref26] Huang G. Liu Z. Van Der Maaten L. Weinberger K. Q. (2017). Densely connected convolutional networks. In *Proceedings of the IEEE conference on computer vision and pattern recognition* (pp. 4700–4708).

[ref27] Jin S. Wang B. Xu H. Luo C. Wei L. Zhao W.. (2020). AI-assisted CT imaging analysis for COVID-19 screening: building and deploying a medical AI system in four weeks. medRxiv [Preprint]. 10.1016/j.asoc.2020.106897 PMC7654325 33199977

[ref28] Karbhari Y. Basu A. Geem Z. W. Han G. T. Sarkar R. (2021). Generation of synthetic chest X-ray images and detection of COVID-19: A deep learning based approach. Diagnostics 11:895. doi: 10.3390/diagnostics11050895, PMID: 34069841 PMC8157360

[ref29] Khalifa N. E. M. Loey M. Taha M. H. N. Mohamed H. N. E. T. (2019). Deep transfer learning models for medical diabetic retinopathy detection. Acta Informatica Medica 27, 327–332. doi: 10.5455/aim.2019.27.327-332, PMID: 32210500 PMC7085308

[ref30] Ksiazek T. G. Erdman D. Goldsmith C. S. Zaki S. R. Peret T. Emery S.. (2003). A novel coronavirus associated with severe acute respiratory syndrome. N. Engl. J. Med. 348, 1953–1966. doi: 10.1056/NEJMoa030781 12690092

[ref31] Lee Y. Kim Y. S. Lee D. I. Jeong S. Kang G. H. Jang Y. S.. (2022). The application of a deep learning system developed to reduce the time for RT-PCR in COVID-19 detection. Sci. Rep. 12, 1–10. doi: 10.1038/s41598-022-05069-2 35075153 PMC8786863

[ref32] Liu W. Jiang Y. Peng L. Sun X. Gan W. Zhao Q.. (2022a). Inferring gene regulatory networks using the improved Markov blanket discovery algorithm. Interdiscipl. Sci. Comput. Life Sci. 14, 168–181. doi: 10.1007/s12539-021-00478-9, PMID: 34495484

[ref33] Liu Z. Lin Y. Cao Y. Hu H. Wei Y. Zhang Z.. (2021). Swin transformer: hierarchical vision transformer using shifted windows. In *Proceedings of the IEEE/CVF International Conference on Computer Vision* (pp. 10012–10022).

[ref34] Liu W. Lin H. Huang L. Peng L. Tang T. Zhao Q.. (2022b). Identification of miRNA-disease associations via deep forest ensemble learning based on autoencoder. Brief. Bioinform. 23:bbac104. doi: 10.1093/bib/bbac104, PMID: 35325038

[ref35] Mofijur M. Fattah I. R. Alam M. A. Islam A. S. Ong H. C. Rahman S. A.. (2021). Impact of COVID-19 on the social, economic, environmental and energy domains: lessons learnt from a global pandemic. Sustain. Product. Consumpt. 26, 343–359. doi: 10.1016/j.spc.2020.10.016, PMID: 33072833 PMC7556229

[ref36] Mustafa B. Loh A. Freyberg J. MacWilliams P. Wilson M. McKinney S. M.. (2021). Supervised Transfer Learning at scale for Medical Imaging. arXiv [Preprint] arXiv:2101.05913.

[ref37] Pan S. J. Yang Q. (2009). A survey on transfer learning. IEEE Trans. Knowl. Data Eng. 22, 1345–1359. doi: 10.1109/TKDE.2009.191

[ref38] Pan F. Ye T. Sun P. Gui S. Liang B. Li L.. (2020). Time course of lung changes on chest CT during recovery from 2019 novel coronavirus (COVID-19) pneumonia. Radiology 295, 715–721. doi: 10.1148/radiol.2020200370, PMID: 32053470 PMC7233367

[ref39] Peters A. Vetter P. Guitart C. Lotfinejad N. Pittet D. (2020). Understanding the emerging coronavirus: what it means for health security and infection prevention. J. Hosp. Infect. 104, 440–448. doi: 10.1016/j.jhin.2020.02.023, PMID: 32145323 PMC7124368

[ref40] Pu R. Liu S. Ren X. Shi D. Ba Y. Huo Y.. (2022). The screening value of RT-LAMP and RT-PCR in the diagnosis of COVID-19: systematic review and meta-analysis. J. Virol. Methods 300:114392. doi: 10.1016/j.jviromet.2021.114392, PMID: 34856308 PMC8629515

[ref41] Radosavovic I. Kosaraju R. P. Girshick R. He K. Dollár P. (2020). Designing network design spaces. In *Proceedings of the IEEE/CVF conference on computer vision and pattern recognition* (pp. 10428–10436).

[ref42] Roberts M. Driggs D. Thorpe M. Gilbey J. Yeung M. Ursprung S.. (2021). Common pitfalls and recommendations for using machine learning to detect and prognosticate for COVID-19 using chest radiographs and CT scans. Nat. Machine Intell. 3, 199–217. doi: 10.1038/s42256-021-00307-0

[ref43] Saad Menezes M. C. Santinelli Pestana D. V. Ferreira J. C. Ribeiro de Carvalho C. R. Felix M. C. Marcilio I. O.. (2022). Distinct outcomes in COVID-19 patients with positive or negative RT-PCR test. Viruses 14:175. doi: 10.3390/v14020175, PMID: 35215772 PMC8874612

[ref44] Sagi O. Rokach L. (2018). “Ensemble learning: A survey,” in Data Mining and Knowledge Discovery, Vol. 8. ed. W. Pedrycz (Hoboken, New Jersey: Wiley Interdisciplinary Reviews) e1249

[ref45] Shalbaf A. Vafaeezadeh M. (2021). Automated detection of COVID-19 using ensemble of transfer learning with deep convolutional neural network based on CT scans. Int. J. Comput. Assist. Radiol. Surg. 16, 115–123. doi: 10.1007/s11548-020-02286-w 33191476 PMC7667011

[ref46] Shorten C. Khoshgoftaar T. M. Furht B. (2021). Deep learning applications for COVID-19. J. Big Data 8, 1–54. doi: 10.1186/s40537-020-00392-9 33457181 PMC7797891

[ref47] Soares E. Angelov P. Biaso S. Froes M. H. Abe D. K. (2020). SARS-CoV-2 CT-scan dataset: A large dataset of real patients CT scans for SARS-CoV-2 identification. medRxiv [Preprint].

[ref48] Sun F. Sun J. Zhao Q. (2022). A deep learning method for predicting metabolite-disease associations via graph neural network. Brief. Bioinform. 23, 1–11. doi: 10.1093/bib/bbac266, PMID: 35817399

[ref49] Sun H. Wang A. Wang L. Wang B. Tian G. Yang J.. (2022a). Systematic tracing of susceptible animals to SARS-CoV-2 by a bioinformatics framework. Front. Microbiol. 13:781770. doi: 10.3389/fmicb.2022.781770 35308363 PMC8931700

[ref50] Tan M. Le Q. (2021). Efficientnetv2: Smaller models and faster training. In *International Conference on Machine Learning* (pp. 10096–10106). PMLR.

[ref51] Ter-Sarkisov A. (2022). Covid-ct-mask-net: prediction of covid-19 from CT scans using regional features. Appl. Intell. 52, 1–12. doi: 10.1007/s10489-021-02731-6 PMC8741555 35035092

[ref52] Vaswani A. Shazeer N. Parmar N. Uszkoreit J. Jones L. Gomez A. N.. (2017). Attention is all you need. Adv. Neural Inf. Proces. Syst. 30, 5998–6008. doi: 10.48550/arXiv.1706.03762

[ref53] Voulodimos A. Doulamis N. Doulamis A. Protopapadakis E. (2018). Deep learning for computer vision: A brief review. Comput. Intell. Neurosci. 2018, 1–13. doi: 10.1155/2018/7068349, PMID: 29487619 PMC5816885

[ref54] Wang C. C. Han C. D. Zhao Q. Chen X. (2021). Circular RNAs and complex diseases: from experimental results to computational models. Brief. Bioinform. 22:bbab286. doi: 10.1093/bib/bbab286, PMID: 34329377 PMC8575014

[ref55] Wang D. Hu B. Hu C. Zhu F. Liu X. Zhang J.. (2020). Clinical characteristics of 138 hospitalized patients with 2019 novel coronavirus–infected pneumonia in Wuhan, China. JAMA 323, 1061–1069. doi: 10.1001/jama.2020.1585, PMID: 32031570 PMC7042881

[ref56] Wang B. Jin S. Yan Q. Xu H. Luo C. Wei L.. (2021). AI-assisted CT imaging analysis for COVID-19 screening: building and deploying a medical AI system. Appl. Soft Comput. 98:106897. doi: 10.1016/j.asoc.2020.106897, PMID: 33199977 PMC7654325

[ref57] Weiss K. Khoshgoftaar T. M. Wang D. (2016). A survey of transfer learning. J. Big Data 3, 1–40. doi: 10.1186/s40537-016-0043-6

[ref58] Worldometer (2022). COVID live - coronavirus statistics - Worldometer. Available at: https://www.worldometers.info/coronavirus/ (Accessed July 11, 2022).

[ref59] Yang M. Yang H. Ji L. Hu X. Tian G. Wang B.. (2022). A multi-omics machine learning framework in predicting the survival of colorectal cancer patients[J]. Comput. Biol. Med. 146:105516. doi: 10.1016/j.compbiomed.2022.105516, PMID: 35468406

[ref60] Zhang J. Xie Y. Li Y. Shen C. Xia Y. (2020). Covid-19 Screening on chest x-ray Images using deep Learning based Anomaly Detection. arXiv [Preprint] arXiv:2003.12338, 27.

[ref61] Zhang L. Yang P. Feng H. Zhao Q. Liu H. (2021). Using network distance analysis to predict lncRNA-miRNA interactions. Interdiscipl. Sci. Comput. Life Sci. 13, 535–545. doi: 10.1007/s12539-021-00458-z, PMID: 34232474

[ref62] Zhao J. Zhang Y. He X. Xie P. (2020). Covid-CT-Dataset: a CT scan Dataset about Covid-19. arXiv [Preprint] arXiv:2003.13865, 490.

[ref63] Zhou T. Lu H. Yang Z. Qiu S. Huo B. Dong Y. (2021). The ensemble deep learning model for novel COVID-19 on CT images. Appl. Soft Comput. 98:106885. doi: 10.1016/j.asoc.2020.106885, PMID: 33192206 PMC7647900

[ref64] Zhou P. Yang X. L. Wang X. G. Hu B. Zhang L. Zhang W.. (2020). A pneumonia Outbreak Associated with a new Coronavirus of Probable bat origin. Nature 579, 270–273. doi: 10.1038/s41586-020-2012-7 32015507 PMC7095418

[ref65] Zhu W. Braun B. Chiang L. H. Romagnoli J. A. (2021). Investigation of transfer learning for image classification and impact on training sample size. Chemom. Intel. Lab. Syst. 211:104269. doi: 10.1016/j.chemolab.2021.104269

[ref66] Zhuang F. Qi Z. Duan K. Xi D. Zhu Y. Zhu H.. (2020). A comprehensive survey on transfer learning. Proc. IEEE 109, 43–76. doi: 10.1109/JPROC.2020.3004555

